# The impact of preoperative patient characteristics on health states after total hip replacement and related satisfaction thresholds: a cohort study

**DOI:** 10.1186/s12955-014-0108-1

**Published:** 2014-08-07

**Authors:** Matthias Vogl, Rainer Wilkesmann, Christian Lausmann, Matthias Hunger, Werner Plötz

**Affiliations:** Helmholtz Zentrum München, German Research Center for Environmental Health, Institute of Health Economics and Health Care Management, P.O. Box 1129, Neuherberg, 85758 Germany; Ludwig-Maximilians-Universität München, Munich School of Management, Institute of Health Economics and Health Care Management & Munich Center of Health Sciences, Munich, Germany; Krankenhaus Barmherzige Brüder München, Akademisches Lehrkrankenhaus der Technischen Universität München, Romanstraße 93, Munich, 80639 Germany; Klinikum rechts der Isar, Technical University Munich, Munich, Germany

**Keywords:** Health-related quality of life, EQ-5D, WOMAC, Total hip replacement, Satisfaction, Patient acceptable symptom state

## Abstract

**Background:**

The aim of the study was to analyze the effect of preoperative patient characteristics on health outcomes 6 months after total hip replacement (THR), to support patient’s decision making in daily practice with predicted health states and satisfaction thresholds. By giving incremental effects for different patient subgroups, we support comparative effectiveness research (CER) on osteoarthritis interventions.

**Methods:**

In 2012, 321 patients participated in health state evaluation before and 6 months after THR. Health-related quality of life (HRQoL) was measured with the EQ-5D questionnaire. Hip-specific pain, function, and mobility were measured with the WOMAC in a prospective observation of a cohort. The predictive capability of preoperative patient characteristics – classified according to socio-demographic factors, medical factors, and health state variables – for changes in health outcomes is tested by correlation analysis and multivariate linear regressions. Related satisfaction thresholds were calculated with the patient acceptable symptom state (PASS) concept.

**Results:**

The mean WOMAC and EQ-5D scores before operation were 52 and 60 respectively (0 worst, 100 best). At the 6-month follow-up, scores improved by 35 and 19 units. On average, patients reported satisfaction with the operation if postoperative (*change*) WOMAC scores were higher than 85 (*32*) and postoperative (*change*) EQ-5D scores were higher than 79 (*14*).

**Conclusions:**

Changes in WOMAC and EQ-5D scores can mainly be explained by preoperative scores. The lower the preoperative WOMAC or EQ-5D scores, the higher the change in the scores. Very good or very poor preoperative scores lower the probability of patient satisfaction with THR. Shared decision making using a personalized risk assessment approach provides predicted health states and satisfaction thresholds.

**Electronic supplementary material:**

The online version of this article (doi:10.1186/s12955-014-0108-1) contains supplementary material, which is available to authorized users.

## Background

Total hip replacement (THR) is an effective operation that relieves pain and improves function, mobility, and health-related quality of life (HRQoL) in patients with osteoarthritis and further diagnoses [1-4]. As hip replacement rates are increasing greatly worldwide [5,6], precise advice for patients with expectancy values for health outcomes and satisfaction thresholds is essential [7]. Thus, besides physician evaluation, a patient-based evaluation of generic and disease-specific health state changes by THR is necessary [4,8]. Therefore, EQ-5D and WOMAC (Western Ontario and McMaster Universities Arthritis Index) questionnaires are recognized as the most reliable, valid, and responsive in the literature [9,10]. Especially in the German context, it is not yet known if this applies to all patient groups and whether these patients are actually satisfied with usually positive health outcomes after THR. Thus, we analyze health outcomes and related satisfaction for the first time in a German prospective observation of a cohort. The current discussion on the benefit of THR can be supplemented by this health economics and medical analysis with patient-based generic quality of life (EQ-5D) and disease-specific function/mobility/pain questionnaires (WOMAC). Patient characteristics and preoperative health scores are used to calculate the patient group-specific, expected value of THR. As our primary objective was to provide patient and clinician support in day-to-day routines of shared decision making, total knee replacement as a comparative procedure was not included in our analysis.

The integrative aspect of health outcome and satisfaction analysis gains importance for two reasons: (1) Shared decision making by patient and clinician – by using a personalized risk assessment approach – is becoming an important challenge to satisfy the patient [11]. This study gives patient group-specific information on average improvements in health outcomes and their time horizon to supply an empirical basis for shared decision making in THR. The study facilitates patient information and supports a patient’s decision making in daily practice by comprehensive measures of health outcomes and satisfaction threshold values. To define the actual value of an intervention for the patient, and make it applicable to the patient, the practitioner, and health policy, an exact knowledge of health outcome drivers is necessary [12]. (2) This study calculates incremental effects for different patient subgroups to inform future intervention studies, cost-effectiveness analyses, modeling approaches, or payment by results in THR. For comparative effectiveness research (CER) on osteoarthritis interventions, a grouping and distribution information for preoperative variables that affect health outcome is generated [13]. Thereby, we question gains in health outcomes for several patient groups.

We used a virtually exhaustive set of patient characteristics that have been shown previously to impact patient outcome in clinical studies [2], as well as information on common side diagnoses, comorbidities, and procedures. There is a research gap on the impact of preoperative patient characteristics on generic health outcomes, useful for understandable patient information and economic analysis of cost-effectiveness and benefit for defined patient groups, e.g., results inform about the probability of and time until a health state is better than before the operation.

The first aim of the study is to analyze the effect of preoperative patient characteristics on postoperative HRQoL, hip-specific pain, and function/mobility to be able to group patients for individual decision making based on the strongest predictors for health outcome changes. This facilitates patient information on surgery outcome and enables economic analysis of total hip replacement (THR) in different patient groups. To evaluate clinically relevant improvements and postoperative states the patient is satisfied with in daily practice, the patient acceptable symptom state (PASS) concept is used [14]. For THR, the PASS concept is a validated instrument in the literature [15,16]. It gives a threshold value beyond which patients define their health state as well or are actually satisfied with the results of THR [14]. 6 months after the operation patients reported health outcomes and were asked whether they were satisfied with THR. Satisfaction results were linked to their postoperative health outcome scores, to be able to support patients in day-to-day routines on their decision on THR with meaningful and appraisable expected health outcome changes from the perspective of the patient, the main objective of this study.

## Patients and methods

### Study design

The study was designed as a single center, prospective observation of a cohort. Dependent variables were the change in HRQoL scores (EQ-5D) and hip-specific pain and function/mobility scores (WOMAC). The study used 6 months for follow-up, as prior studies have shown that most health improvements are reached within this period [15]. The 6-month time horizon is more imaginable and meaningful for a shared decision making situation with the patient than a later follow-up. However, this implies that the patient should be informed that the scores slightly underestimate the improvements by THA after a year or even three years as especially the function scores will still slightly improve after the 6-month time horizon. Predictive patient characteristics analyzed were separated into three subdomains: (1) *socio-demographic factors*; (2) *medical factors*; (3) *HRQoL, pain, function, and mobility before THR* (Table 1). This virtually exhaustive set of patient characteristics has been shown previously to impact patient outcome in clinical studies [2]. Essential for the observational study design was that all patients were treated similarly according to their major diagnosis, independent of their preoperative characteristics [17].

**Table 1:** **Predictive patient characteristics**

Significant Spearman’s rank correlation (p < 0.05): relation of patient characteristics to WOMAC and EQ-5D VAS score *preoperative ^change °postoperative	**Frequency/mean (SD)**	**%**
**Side diagnoses, at least 10 times in study patients**		
D62 - acute anemia*^°	25	8.5
E03 - hypothyroidism	47	16.7
E11 - diabetes*°	23	8.2
E66 - obesity*^°	17	6.0
E78 - lipidemia	27	9.6
E79 - purine/pyrimidine metabolism	13	4.6
E86 - hypovolemia	13	4.6
E87 - dysfunction of water/electrolyte balance	17	6.0
F32 - depression	11	3.9
I10 - arterial hypertonicity	155	55.2
I25 - ischemic heart disease	15	5.3
I48 - atrial fibrillation	10	3.6
J45 - asthma	11	3.9
N18/N39 - renal failure and related diseases	17	6.1
Z88 - drug allergy*	14	5.0
Z91 - risk factors in personal anamnesis*	14	5.0
Z92 - care of personal anamnesis°	39	13.9
Z95 - cardiac/vascular implants	23	8.2
Z96 - other functional implants°	42	14.9
n.n. - cardiopathy	25	8.9
n.n. - COPD	11	3.9
n.n. - hypercholesterolemia	41	14.6
n.n. - myocardial infarction/stent	18	6.4
n.n. - reflux^	17	6.0
n.n. - major hip distortion^	20	7.2
n.n. - deep venous thrombosis (DVT)	12	4.3
Number of side diagnoses*°	3.6 (2.9)	
**Operations and procedures, at least 5 times in study patients**		
5-791 - open reposition of fracture	5	1.8
5-829 - other arthroplasty*^	8	2.8
5-986 - minimally invasive technique	270	96.1
8-919 - acute pain relief	5	1.8
8-930/1 - monitoring	26	9.3
Number of operations and procedures*	2.4 (1.1)	
**Other**		
Age°	67.7 (10.1)	
Gender male*^°	117	41.6
BMI*	26.9 (4.9)	
≥ 30*^°	48	17.5
Major diagnosis°		
Coxarthrosis	279	99.3
Osteonecrosis	2	0.7
Operations at joint before procedure°		
0	263	93.9
1	12	4.3
2 or more	5	1.8
Marital status*^°		
Married	184	65.9
Single	25	9.0
Divorced/living apart	32	11.4
Widowed	38	13.6
Housing situation*°		
Alone	79	28.4
With partner	141	50.2
With family	58	20.9
Discharge home (others inpatient rehabilitation)°	53	18.8
Health insurance compulsory (others private)*°	136	48.4
Already THR°	58	20.6
Preoperative hemoglobin*^°	14.1 (1.2)	
Blood transfusion (yes)^	13	4.7
ASA score*°		
1	109	38.8
2	150	53.4
3 or higher	22	7.9
Charlson Comorbidity Index*^°		
0	210	74.4
1	55	19.6
2	9	3.2
3 or higher	7	2.5
Metabolic syndrome (yes)*°	8	2.7
Cement (cement or hybrid)*	33	11.8

Recent studies refer to minimum clinically important differences (MCID) or patient acceptable symptom state (PASS) to define clinically relevant states for the patient [7,14,18,19]. As we want to define cut-off values of WOMAC or EQ-5D scores that are associated with patient satisfaction in THR and not only with a significant change in WOMAC or EQ-5D scores, two well established PASS methods were used: to calculate beyond which health state the patients are satisfied with THR outcome (“Were your expectations on THR fulfilled?”), we calculated PASS scores with the 75th centile method and the receiver operating characteristic (ROC) curve method [20]. Thereby, PASS is an anchor to provide more meaningful patient information and to help in interpreting health state results for patients and clinicians.

### Study population

**Figure 1:**
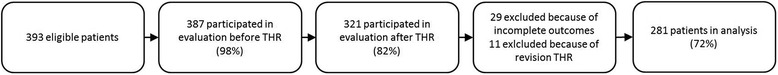
**Flow chart on patients included.**

From January 2012 to June 2012, 387 patients at BBM Clinic participated in the health state evaluation before THR at the admission day and 321 also participated in the follow-up evaluation 6 months after discharge (Figure 1). Patients lost to follow-up did not differ significantly in most baseline characteristics. However, they had a statistically significantly lower preoperative WOMAC score (−5.2) and EQ-5D score (−3.4). There were no exclusion criteria except missing patient consent. Besides patients with the major diagnosis of osteoarthritis of the hip, the study also included individuals with osteonecrosis. As we excluded revision THR, we had no mechanical complications or infections for major diagnoses. The study had approval from the ethics commission of Klinikum rechts der Isar, Technical University Munich.

### Measuring instruments

According to clinical practice guidance, pain, function/mobility, and HRQoL are limitations related to major diagnoses leading to THR [21,22]. We measured HRQoL with the EQ-5D, a generic instrument that generates an index value with a standard formula out of five dimensions: mobility, self-care, usual activity, pain/discomfort, and anxiety/depression [23]. Each dimension has three possible levels to report current problems: no problems, some problems, and severe problems. To assign an overall value to each of the possible 253 health states, two standardized formulas are used [23,24]. To enable comparison with former studies and provide a basis for modeling purposes (e.g., for QALY calculation), we provide HRQoL outcomes based on the widely used UK population preference-weighted EQ-5D index values [24,25]. To respect actual experiences in the German population, we use a recently developed German population experience-weighted EQ-5D index [23]. The EQ-5D VAS that measures the overall health state was completed by the study population as the gold standard. The EQ-5D score is a qualified and well recognized health outcome measure in patients who receive THR: it has been tested for its validity, reliability, and responsiveness in relation to the SF-36, SF-6D, WOMAC, and Oxford Hip Score [9,26].

To measure hip-specific outcome from the patients’ view, we use the WOMAC with its three subscales on pain (five sub-questions), stiffness (two sub-questions), mobility (17 sub-questions), and an overall score based on the three subscales. The sub-questions used a Likert scale from 0 to 10. The questionnaire showed the best psychometric characteristics for hip and knee replacement patients in disease-specific questionnaires [10] and has also been tested for its validity in the German version [27,28]. We normalized each score into a 0–100 scale, with 0 being the poorest and 100 being the best possible score, to improve comparability. Satisfaction with THR was measured using a Likert scale from 0 to 10, with 9 and 10 defined as satisfied with THR. In the following we additionally provide change scores besides pre- and postoperative scores to support patient comprehension in day-to-day routines.

### Statistical analysis

We provide descriptive statistics on the distribution of postoperative scores and corresponding change scores, and examined bivariate association with preoperative patient characteristics, using Spearman’s rank correlation. In case Spearman’s correlation is significant (p < 0.05), variables were considered as potential covariates for multivariate ordinary least squares (OLS) regression analyses. Statistical significance was assumed with p < 0.05. A power calculation (alpha = 0.05) for repeated measures of WOMAC and EQ-5D analyses showed an observed power of 1. The final set of covariates was determined using a backward selection method based on significance in regression analysis. When comparing the health outcomes of preoperative EQ-5D and WOMAC groups, it has to be borne in mind that we use an observational study design where a regression to the mean effect is possible [29].

**Figure 2:**
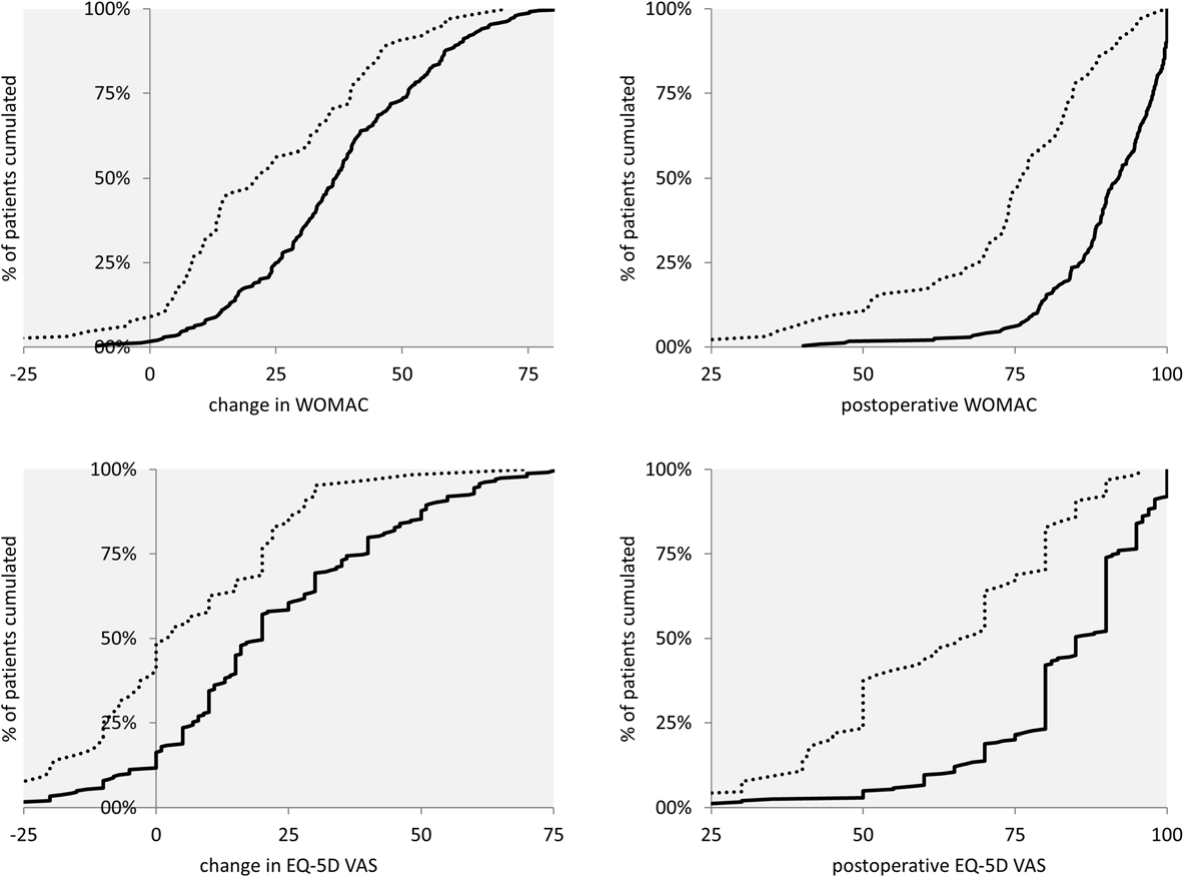
**Distribution function to determine patient acceptable symptom state (PASS) with 75th centile method.** Figure legend: **∙∙∙∙∙** unsatisfied patients **──** satisfied patients.

**Figure 3:**
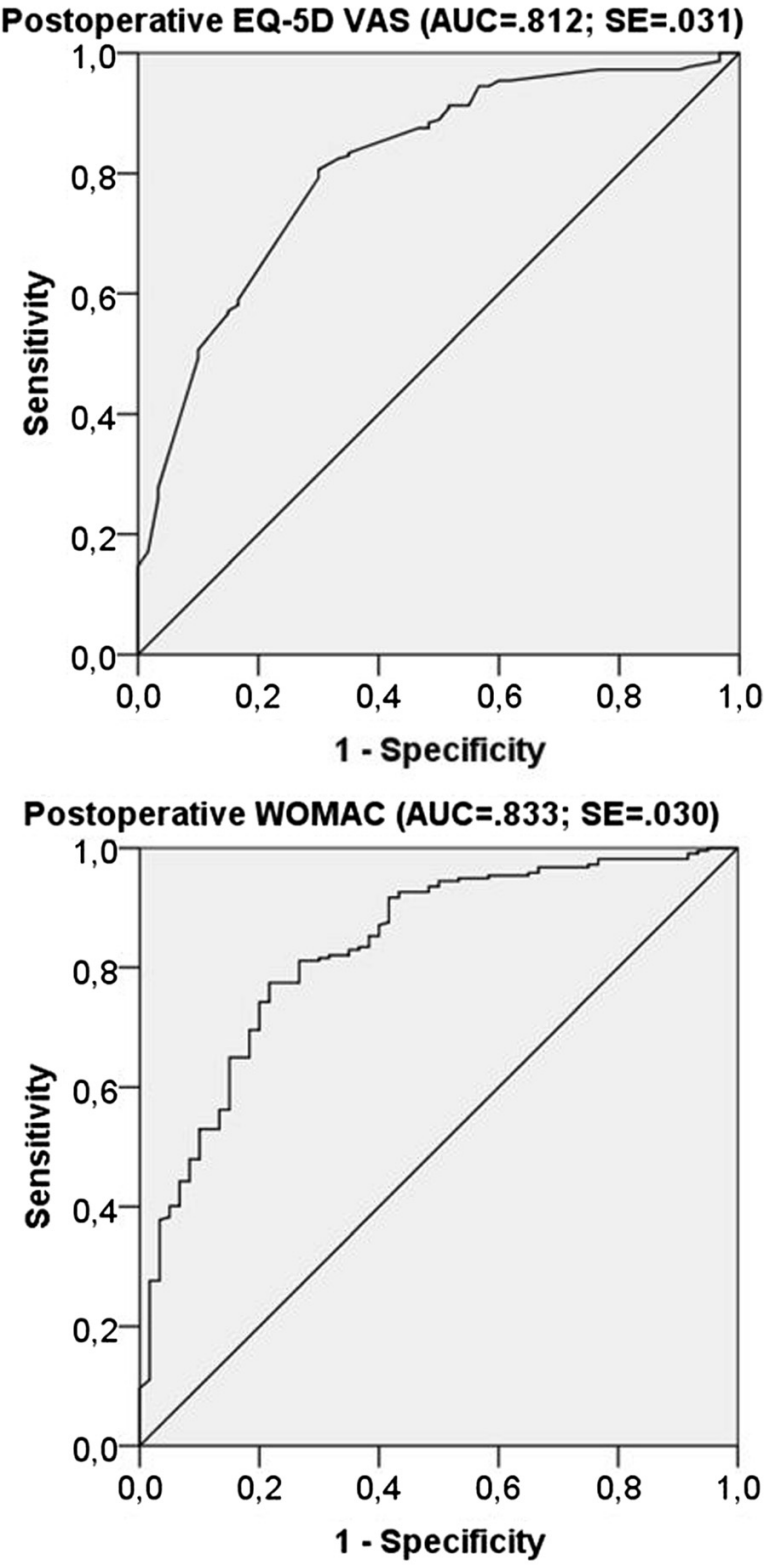
**ROC curve using satisfaction and postoperative WOAMC/EQ-5D VAS scores.**

In determining clinically relevant health state changes (PASS), we were especially interested in the very satisfied group, as these estimates are not yet available and literature demands cut-off values for the highest levels of satisfaction [30] (Figure 2 and Figure 3). The ROC curve was used to identify satisfaction cut-off values for changes and 6-month postoperative WOMAC and EQ-5D scores. The ROC curve displays sensitivity/specificity pairs, where each corresponds to a possible cut-off value. We defined the optimal cut-off value where the difference in sensitivity and specificity is minimized. The area under the ROC curve (AUC) measures how well preoperative EQ-5D and WOMAC values separate between satisfied and unsatisfied patients. The closer the AUC is to 1 (the better it fills the upper triangles in Figure 3), the better EQ-5D and WOMAC can distinguish between satisfied and unsatisfied patients. Distribution functions of satisfied and unsatisfied patients were generated to show at which WOMAC or EQ-5D state their functions differentiate and to validate ROC curve results [16,20] (75th centile method). The 75th centile method calculates health state values reached by 75% of the satisfied/unsatisfied patients (Figure 2).

## Results

### Health outcomes

The average age of patients was 68 years, 58% were female, and most patients had few general diseases (ASA 1 or 2 and Charlson Comorbidity Index 0 or 1). Some 21% of the patients already had a hip replacement at the opposite side. Table 1 gives an overview of all control variables: significant associations with the changes and postoperative WOMAC and EQ-5D scores were observed for acute anemia, major hip distortion, BMI, preoperative hemoglobin, Charlson Comorbidity Index, etc. (Table 1).

**Figure 4:**
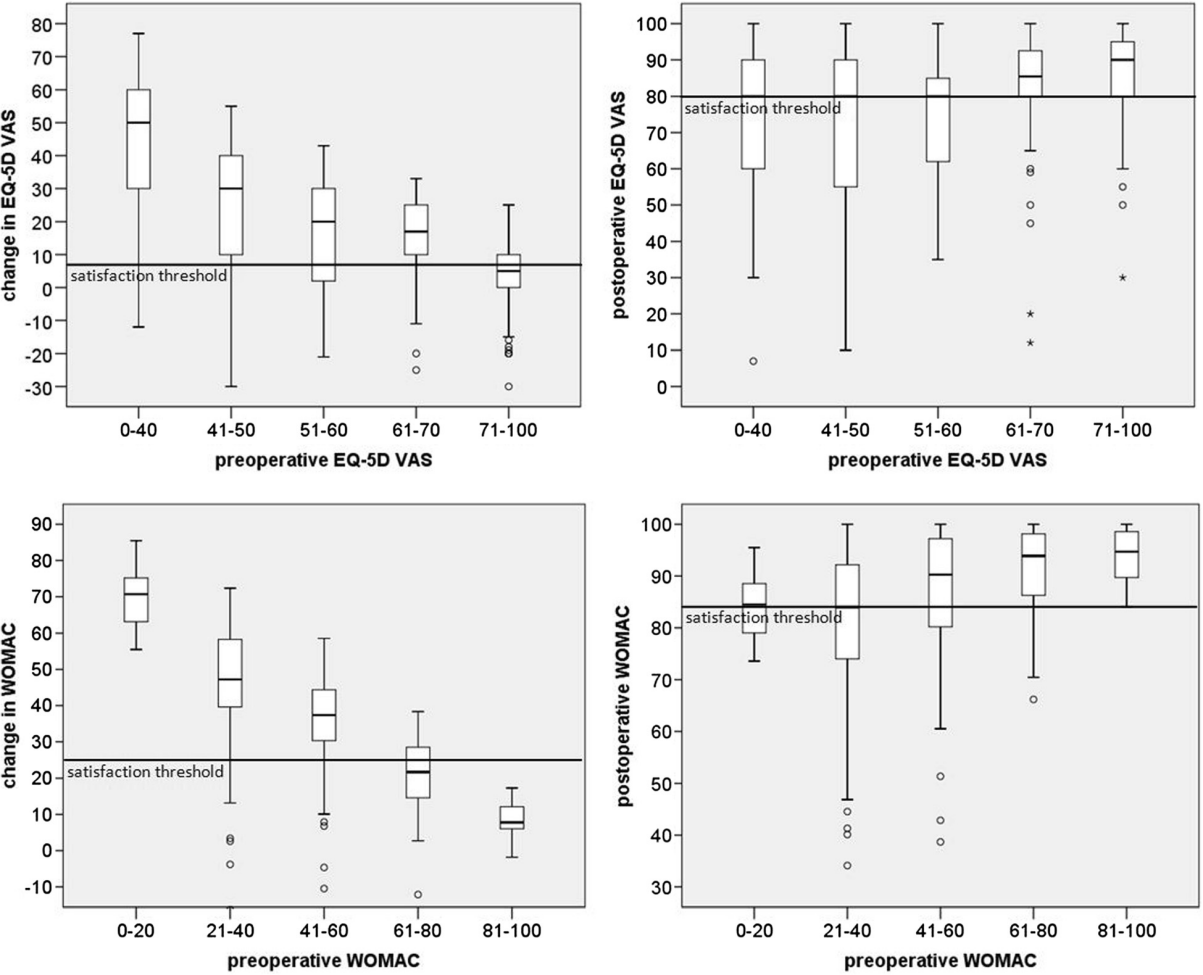
**WOMAC sum and EQ-5D VAS boxplots.** The horizontal lines correspond to the satisfaction thresholds (75th centile method). Satisfaction is assumed at a 9 or 10 on a 0–10 satisfaction Likert scale.

Variance in health outcome changes was mainly explained by preoperative WOMAC and EQ-5D scores. A correlation matrix has shown high correlation of preoperative scores with score changes, 6-month follow-up scores, and weeks until a health state better than preoperative was achieved. Average WOMAC score and EQ-5D VAS before operation were 52 and 60 (0 worst, 100 best). Six months after THR, they had improved by 35 and 19 units (Figure 4 and Table 2). WOMAC and EQ-5D subscales improved accordingly. The poorer the preoperative WOMAC or any EQ-5D score, the higher the change in the scores. Patients with still acceptable preoperative scores had only slightly better postoperative scores compared with patients with poor preoperative WOMAC and EQ-5D scores (Figure 4 and Table 2). On average, 2% of THR patients described their health state, pain, and mobility as worse than before the procedure, about 6% described it as similar, 29% as better, and 63% as much better (Table 3). This description is highly correlated with WOMAC and all EQ-5D scores. Within EQ-5D dimensions, especially pain/discomfort, usual activity, and mobility improved for most patients (Additional file 1). On average, it takes 10 weeks until health state or daily routine is better than before the operation, 5 weeks until pain is better, and 8 weeks until mobility is better than before the operation (Table 3). The time until daily routine is better than before the operation decreases with a high preoperative WOMAC or EQ-5D score. With the patient characteristics analyzed, it was impossible to predict the time until pain or mobility was better than before the operation for different subgroups.

**Table 2:** **Changes in WOMAC and EQ-5D**

**0 worst, 100 best**	**Pre-operative**		**6 months follow-up**		**Change**		
**Preoperative**	**Mean**	**SE**	**Mean**	**SE**	**Mean**	**SE**	**N**
**WOMAC pain**	**55.6**	**1.1**	**90.4**	**0.8**	**34.7**	**1.2**	**281**
0-20	12.9	1.3	89.0	2.1	76.1	2.2	14
21-40	33.1	0.8	85.7	2.4	52.6	2.6	45
41-60	51.4	0.5	87.8	1.4	36.4	1.4	110
61-80	70.8	0.6	94.9	0.8	24.1	1.0	89
81-100	87.7	1.0	95.3	1.2	7.7	1.6	23
**WOMAC stiffness**	**46.6**	**1.4**	**83.0**	**1.0**	**36.5**	**1.5**	**281**
0-20	14.1	0.8	79.1	2.5	65.0	2.7	56
21-40	33.2	0.7	81.1	2.2	48.0	2.3	66
41-60	53.4	0.6	82.2	2.0	28.8	2.0	86
61-80	71.9	0.8	87.8	1.6	15.8	1.9	60
81-100	91.9	1.3	93.1	3.1	1.2	2.5	13
**WOMAC function**	**51.3**	**1.1**	**86.7**	**0.8**	**35.5**	**1.1**	**281**
0-20	16.4	1.0	79.4	3.1	63.4	3.4	21
21-40	33.6	0.6	78.8	2.1	45.3	2.2	69
41-60	50.5	0.6	88.1	1.2	37.5	1.3	96
61-80	69.4	0.6	92.2	0.8	22.8	1.1	77
81-100	85.9	1.0	94.9	1.2	9.0	1.3	18
**WOMAC sum**	**51.8**	**1.1**	**86.7**	**0.8**	**34.9**	**1.1**	**281**
0-20	15.2	1.4	84.2	1.7	69.0	2.2	14
21-40	33.7	0.6	79.6	2.1	45.9	2.2	71
41-60	51.2	0.6	87.4	1.2	36.2	1.3	108
61-80	70.3	0.7	91.6	1.0	21.3	1.2	73
81-100	85.4	0.9	93.9	1.3	8.5	1.3	15
EQ-5D index*	**53.9**	**0.9**	**76.9**	**0.9**	**22.9**	**1.0**	**281**
0-40	33.1	0.5	70.1	2.1	37.1	2.1	69
41-50	46.8	0.3	76.2	2.2	29.3	2.1	41
51-60	54.2	0.2	76.4	1.7	22.2	1.8	70
61-70	65.5	0.3	80.7	1.4	15.3	1.5	53
71-100	76.9	0.6	83.5	1.4	6.7	1.6	48
EQ-5D index**	**51.4**	**1.8**	**84.7**	**1.1**	**33.3**	**1.8**	**281**
−40	8.8	1.1	79.4	2.5	70.5	2.3	88
41-50	-	-	-	-	-	-	-
51-60	56.8	0.6	85.4	3.3	28.6	3.0	26
61-70	66.6	0.3	84.3	1.8	17.7	1.9	73
71-100	77.9	0.6	89.8	1.3	12.0	1.4	94
**EQ-5D VAS**	**59.6**	**1.2**	**78.4**	**1.1**	**18.8**	**1.4**	**281**
0-40	28.0	1.3	74.7	2.8	46.7	2.6	57
41-50	48.3	0.4	70.1	3.4	21.8	3.4	49
51-60	58.4	0.5	74.1	2.8	15.7	2.9	37
61-70	68.8	0.3	81.5	2.5	12.8	2.6	52
71-100	81.9	0.8	85.4	1.3	3.5	1.3	86

*German population experience-weighted.

**UK population preference-weighted.

**Table 3:** **Weeks until effect was reached and general effect 6 months postoperatively**

**Weeks until effect was reached**	Daily routine		Pain		Mobility	
Weeks mean	9.6		5.1		8.1	
Weeks SD	5.4		4.9		5.2	
Not yet better	6.0%		4.9%		5.6%	
**Effect 6 month postoperatively**	Frequency	%	Frequency	%	Frequency	%
Much worse	0	0.0	0	0.0	3	1.1
Worse	7	2.6	3	1.1	5	1.8
Similar	21	7.7	7	2.6	18	6.6
Better	90	32.8	50	18.3	98	35.8
Much better	156	56.9	213	78.0	150	54.7

After multivariate regression analyses, we see that about 68% of the variance of changes in WOMAC and 47% of the variance of changes in EQ-5D VAS can be explained by very few preoperative patient characteristics: preoperative WOMAC and EQ-5D VAS scores, ASA score, metabolic syndrome, etc. (Table 4). When performing multivariate analysis with and without preoperative scores, we see that 75% of the explained variance is explained by the preoperative WOMAC score in the WOMAC change score model, and for the EQ-5D VAS change score model, 81% of the explained variance is explained by the preoperative EQ-5D VAS score. Thus, the change in WOMAC and EQ-5D can be well explained by preoperative WOMAC and EQ-5D only. Special risk groups or patient groups in which THR had no positive effect on WOMAC or EQ-5D scores could not be detected. Based on our statistical analyses, patients who do not benefit from THR cannot be identified by preoperative socio-demographic, medical, and health state characteristics.

**Table 4:** **Multivariate linear regression**

**Preoperative values**	**Change in WOMAC sum**		**Change in EQ-5D VAS**	
*p < .05 **p < .01				
	**(adj.R** ^**2**^ **= 0 .68)**		**(adj.R** ^**2**^ **= 0.47)**	
	**Coefficient**	**SE**	**Coefficient**	**SE**
Constant	**98.307	6.455	**77.959	10.141
WOMAC pain score	**-0.166	.062	-.189	.099
WOMAC stiffness score	**-0.191	.042		
WOMAC function score	**-0.536	.068	.185	.101
EQ-5D usual activity severe problems compared to none	**-47.200	11.407		
EQ-5D anxiety/depression some problems compared to none	**-4.454	1.598	*-5.820	2.563
EQ-5D visual analog scale (VAS)			**-0.797	.064
major hip distortion	−4.706	2.797	−8.306	4.462
E11 - diabetes	−6.033	3.108		
E66 - obesity	5.735	3.247		
Z96 - other functional implants	*-3.896	1.946		
5-829 - other arthroplasty			−14.422	7.363
Reflux	**-8.502	2.901		
Number of operations and procedures	−1.277	.762		
Housing situation family compared to alone	−18.877	11.151		
Discharge home compared to inpatient rehabilitation	3.537	1.831		
ASA 2 compared to 1	**-4.611	1.501		
Discharge home compared to inpatient rehabilitation			*7.113	2.789
Metabolic syndrome	**-13.752	5.147	**-22.776	6.432

### Satisfaction outcomes

PASS estimates based on the ROC curve method and the 75th centile method were very similar. ROC curve estimates (Figure 3) of patients who considered their health state as satisfactory were above 85 for postoperative WOMAC (sensitivity 77%, specificity 77%), above 32 for change in WOMAC, above 79 for postoperative EQ-5D VAS (sensitivity 79%, specificity 70%), and above 14 for change in EQ-5D VAS. Sensitivity analyses on the satisfaction Likert scale, calculating cut-off values including 8, 9, and 10 or only 10 as the satisfactory state, show stable cut-off values. When only 10 is measured as satisfactory, cut-off values for 6-month postoperative states are 2 points higher; when 8 is included, cut-off values do not change. 75th centile estimates show similar results: of patients who considered their state satisfactory, 75% had a change in WOMAC of more than 25 (CI ±2). The satisfied patients had a postoperative WOMAC score above 86 (CI ±1). Concerning EQ-5D VAS change, 75% of the satisfied patients noted a change of 8 or higher (CI ±3). They had a postoperative EQ-5D VAS state of 80 or higher (CI ±3) (Figure 2).

Socio-demographic and medical covariates for satisfied and unsatisfied patients did not vary with PASS values of both WOMAC and EQ-5D VAS, while preoperative WOMAC and EQ-5D VAS scores correlated with PASS (p < 0.01) [20,30]. PASS outcomes show low probability of satisfaction for patients with WOMAC changes of less than 25 and EQ-5D VAS changes of less than 8. These patients have average preoperative WOMAC and EQ-5D VAS scores of 67 and 73 compared with 45 and 54 for the satisfied patients. Patients with postoperative WOMAC below 86 and EQ-5D VAS below 80 also showed low probability of satisfaction. These patients have average preoperative WOMAC and EQ-5D VAS scores of 45 and 51 compared with 56 and 64 for the satisfied patients. This suggests that health outcome and preoperative health scores are related to satisfaction after THR: very poor and very good preoperative scores correlate with low satisfaction.

## Discussion

### Health outcomes

This study contributes to the literature of predictors of postoperative health outcomes after THR and the literature on predicting satisfaction after THR by investigating both subjects integrative in the German context. Related studies are by trend conform to our study [3,7,31-33]. THR outcome was mainly related to preoperative WOMAC and EQ-5D scores. There was a high correlation between WOMAC and EQ-5D scores, showing that the WOMAC results of THR patients can explain large parts of HRQoL, and that EQ-5D is a responsive instrument for THR patients. Although socio-demographic and medical covariates showed correlation with change and postoperative WOMAC and EQ-5D scores (Table 1), their contribution to the explanation was very low in multivariate models once the preoperative WOMAC and EQ-5D scores are accounted for (Table 4). Other studies were inconclusive on the impact of other preoperative variables than health state scores on postoperative health outcomes [2,3]. That predictability is highest with preoperative scores is in accordance with studies by Fortin et al., Röder et al., and Hawker et al. [7,32,34].

The separation of the most predictive variables (WOMAC and EQ-5D preoperatively) to support individual patient information and decision making showed that, for all groups, a significant improvement in WOMAC and HRQoL scores can be reached. The improvement is lowest in the group with still acceptable preoperative WOMAC scores (81–100) and acceptable preoperative EQ-5D scores (71–100). According to clinical practice guidance, conservative therapy is useful when pain is still low and there are minor limitations in function/mobility and HRQoL. Afterwards there is an optimal time slot for THR where health outcomes and satisfaction are best. This conforms with the study results showing that patients with still acceptable preoperative WOMAC and EQ-5D scores have a tendency to benefit less from THR and also have a lower probability of being satisfied, as possible improvements from THR are lower (Figure 4). For patients with poorest scores, who also have a tendency to benefit less from THR, conservative therapy is no longer an option, questioning the use of THR only for patients in the group of still acceptable WOMAC or EQ-5D scores.

### Satisfaction outcomes

In direct comparison to PASS results by Anakwe et al., Escobar et al., and Kvamme et al., we calculated a higher satisfaction threshold for EQ-5D and WOMAC scores as our baseline and follow-up scores of WOMAC and EQ-5D were higher [15,35,36]. Anakwe et al. showed that unlike postoperative scores, preoperative function scores are not related to satisfaction [35]. Several other studies found that WOMAC [7,15,16,35,36] and EQ-5D [36] postoperative scores can be related to a PASS. As a prediction of PASS satisfaction measures to support patient’s choice on THR does not yet exist, we used an indirect way to predict satisfaction for the patient preoperatively: we related satisfaction measures to postoperative and change scores that can be expected by the classification of a patient into the given preoperative WOMAC and EQ-5D score groups.

The classification into preoperative EQ-5D and WOMAC groups and related satisfaction thresholds allows informed decision making on THR when included in preoperative patient information [30]. Presenting the patient’s expected WOMAC and EQ-5D outcomes in a boxplot, separated by preoperative scores can facilitate patient choice when preoperative WOMAC and EQ-5D scores are calculated for the patient (Figure 4 and Additional file 2). In this study, satisfaction thresholds are given for the first time for the highest levels of satisfaction, and for WOMAC and EQ-5D changes and 6-month follow-up states. Besides this patient information, the introduction of standardized performance measures from a patient’s perspective also has management relevance concerning marketing at the patient or practitioner level.

### Strengths and weaknesses

Strengths of the study are the large patient group reached in a short period in a single hospital – allowing a distribution of the predictive factors – and the nearly exhaustive set of control variables analyzed simultaneously. Modelers benefit from preoperative EQ-5D and WOMAC distribution information. The distribution information on WOMAC and EQ-5D (Table 4) enables a transformation of WOMAC values into EQ-5D values, allowing (cost-)utility calculations with quality-adjusted life years (QALY) for studies that used WOMAC only. Another advantage is the conduct of the study in a standard supply hospital with a large catchment area to get a cross-section of patients and avoid preselection concerning, e.g., comorbidity [37]. On the other hand, generalizability of study results might not be given, as we used a single hospital to include only patients treated identically according to their medical need. Where applicable, patients were operated with minimally invasive technique. With patients needing uncemented arthroplasty the Allofit cup/Spotorno stem system by Zimmer was used as the standard, for hybrid systems a similar cup and a Müller Straight stem by Zimmer was used, and for cemented systems a Müller PE cup and a Müller Straight stem was used as the standard. Case numbers for other arthroplasty techniques than minimally invasive or for prosthesis types were too low in the subgroups to find statistically significant differences.

Weaknesses of the study are: (1) the low patient number in some subgroups that did, e.g., not allow to go into a further detailed analysis of operation techniques and prosthesis type used; (2) that socio-economic variables were limited to compulsory and private health insurance, differentiating roughly between high and low income patients. Here we would expect differences in socio-economic groups, as a difference in health outcome between compulsory and private insurance could already be detected; (3) that more patients with lower a health state did not participate in follow-up, although a lower preoperative health state in WOMAC and EQ-5D is related to a higher than average health improvement; (4) p-values in Table 4 are of restricted validity as we had to specify and estimate on the same dataset – a split-sample design was not reasonable due to the limited number of cases and would have supported validity of results; and (5) we did not calculate special PASS values for patients in each preoperative WOMAC and EQ-5D index score group besides the overall PASS score, as we did not have enough observations in each category to calculate a special PASS value in each group with either the ROC curve method [16] or the 75th centile method [14]. By getting similar PASS cut-off values with both methods and similar cut-off values within a sensitivity analysis for the satisfaction measure, we expect to have robust results for the satisfaction thresholds of WOMAC and EQ-5D. However, as patients with different preoperative health states might have different perceptions on their satisfaction threshold, this subgroup analysis should be researched in the future.

### A future perspective

Change and postoperative HRQoL and WOMAC scores are useful performance or patient value measures that can be used as quality indicators in pay for performance systems [12]. In case WOMAC and EQ-5D change scores are used as additional influencing factors for performance-related reimbursement, the WOMAC is the preferred instrument, as it is less influenced by comorbidities not related to THR and has higher correlation with the preoperative score. Future research should match pre- and postoperative EQ-5D values with population normative values to further quantify the utility of THR for each preoperative EQ-5D group. The study allows a combination of HRQoL measures with costing data at patient level, supporting national and international comparison of effects and cost-effectiveness based on preoperative EQ-5D scores. By giving incremental effects for different patient subgroups, we support future comparative effectiveness research on osteoarthritis interventions and across countries. The National Health Service (NHS) in England has already introduced the EQ-5D questionnaire as a standardized questionnaire for a patient-reported outcome measure (PROM), to be able to measure hospital performance and cost-effectiveness for patient groups. A comparison of EQ-5D changes with English values [38] on a patient basis can show how routine data collection of health outcomes can be introduced in further countries. To provide patients not only with short term expectations (6 months) on health outcomes, quality of life, and satisfaction, a second follow-up period at 24 or 36 month should be provided for long term expectations as especially function scores still slightly improve after 6 months [2].

## Conclusions

Changes in WOMAC and EQ-5D scores can mainly be explained by preoperative scores. Other covariates contributed only marginally to predict THR health outcomes. Based on the PASS concept, very good or very poor preoperative scores lower the probability of patient satisfaction with THR. However, a patient group in which HRQoL or WOMAC scores could not be improved or a patient group that was unsatisfied could not be detected. THR should not be restricted to subgroups, as all subgroups can benefit. Shared decision making using a personalized risk assessment approach provides predicted health states and satisfaction thresholds to patients and can support the individual decision on THR.

## Additional files

Additional file 1:
**Changes in EQ-5D dimensions.**
Additional file 2:
**Slide on expected WOMAC and EQ-5D outcomes for German patients.**

## Abbreviations

ASA: American Society of Anesthesiologists
CER: Comparative effectiveness research
EQ-5D: EuroQol 5-dimension
EQ-5D VAS: EuroQol 5-dimension visual analogue scale
HRQoL: Health-related quality of life
OLS: Ordinary least squares
THR: Total hip replacement
PASS: Patient acceptable symptom state
PROMs: Patient-reported outcome measures
NHS: National Health Service
QALY: Quality-adjusted life year
ROC: Receiver operating characteristic
WOMAC: Western Ontario and McMaster Universities Arthritis Index
