# Supplementary material for: The impact of preoperative patient characteristics on health states after total hip replacement and related satisfaction thresholds: a cohort study
Source: Health Qual Life Outcomes. 2014 Aug 7;12:108. doi: 10.1186/s12955-014-0108-1 (PMC4159538; doi:10.1186/s12955-014-0108-1)
Supplement: Additional file 1: — Changes in EQ-5D dimensions. [file 12955_2014_108_MOESM1_ESM.doc]

**Additional file 1: Changes in EQ-5D dimensions**

| EQ-5D mobility | 6 months follow-up | | | | |
| --- | --- | --- | --- | --- | --- |
|  |  | no | some | severe | total |
| before procedure | no | 80 | 6 | 0 | 86 |
|  | some | 142 | 53 | 0 | 195 |
|  | severe | 0 | 0 | 0 | 0 |
|  | total | 222 | 59 | 0 | 281 |
|  |  |  |  |  |  |
| EQ-5D self-care | 6 months follow-up | | | | |
|  |  | no | some | severe | total |
| before procedure | no | 214 | 3 | 0 | 217 |
|  | some | 48 | 15 | 0 | 63 |
|  | severe | 1 | 0 | 0 | 1 |
|  | total | 263 | 18 | 0 | 281 |
|  |  |  |  |  |  |
| EQ-5D usual activity | 6 months follow-up | | | | |
|  |  | no | some | severe | total |
| before procedure | no | 91 | 6 | 0 | 97 |
|  | some | 124 | 53 | 1 | 178 |
|  | severe | 4 | 2 | 0 | 6 |
|  | total | 219 | 61 | 1 | 281 |
|  |  |  |  |  |  |
| EQ-5D pain/discomfort | 6 months follow-up | | | | |
|  |  | no | some | severe | total |
| before procedure | no | 4 | 4 | 0 | 8 |
|  | some | 106 | 80 | 0 | 186 |
|  | severe | 41 | 42 | 4 | 87 |
|  | total | 151 | 126 | 4 | 281 |
|  |  |  |  |  |  |
| EQ-5D anxiety/depression | 6 months follow-up | | | | |
|  |  | no | some | severe | total |
| before procedure | no | 182 | 9 | 0 | 191 |
|  | some | 54 | 30 | 3 | 87 |
|  | severe | 2 | 1 | 0 | 3 |
|  | total | 238 | 40 | 3 | 281 |
