# Supplementary material for: The impact of preoperative patient characteristics on health states after total hip replacement and related satisfaction thresholds: a cohort study
Source: Health Qual Life Outcomes. 2014 Aug 7;12:108. doi: 10.1186/s12955-014-0108-1 (PMC4159538; doi:10.1186/s12955-014-0108-1)
Supplement: Additional file 2: — Slide on expected WOMAC and EQ-5D outcomes for German patients. [file 12955_2014_108_MOESM2_ESM.doc]

**WOMAC (Schmerzen, Steifigkeit und Beweglichkeit/Funktion) 6 Monate nach Hüftoperation**

**
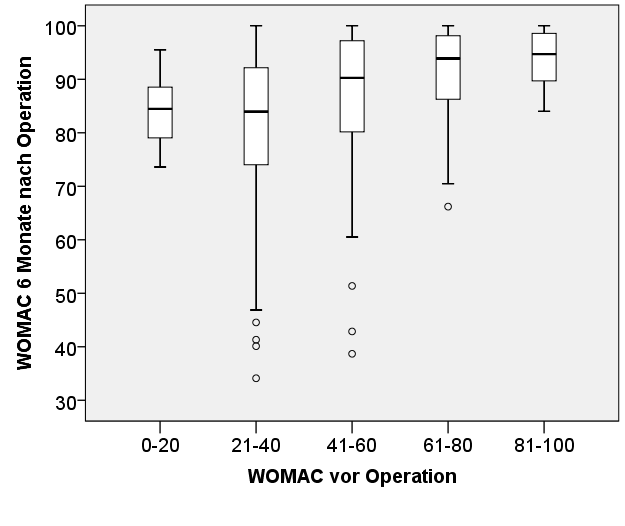

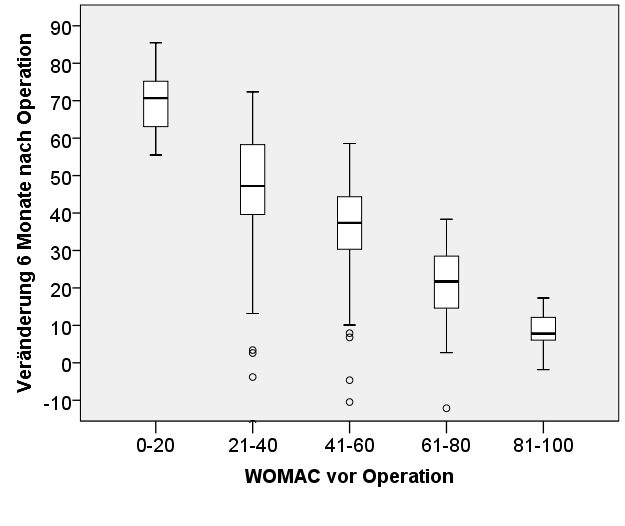
**

**Zufriedenheitsschwelle**

**Zufriedenheitsschwelle**

**Zufriedenheitsschwelle:**

**Zufriedenheit mit dem Ergebnis der Operation 6 Monate nach OP wenn auf einer Skala von 0 - 10 entweder 9 oder 10 angekreuzt wird.**

**Lebensqualität 6 Monate nach Hüftoperation**

**
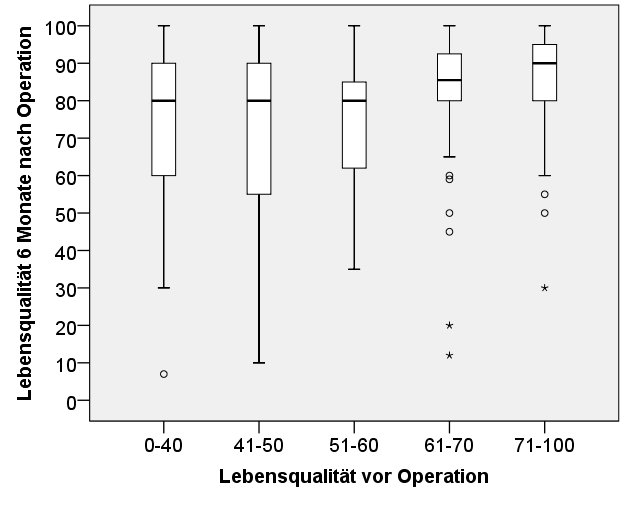

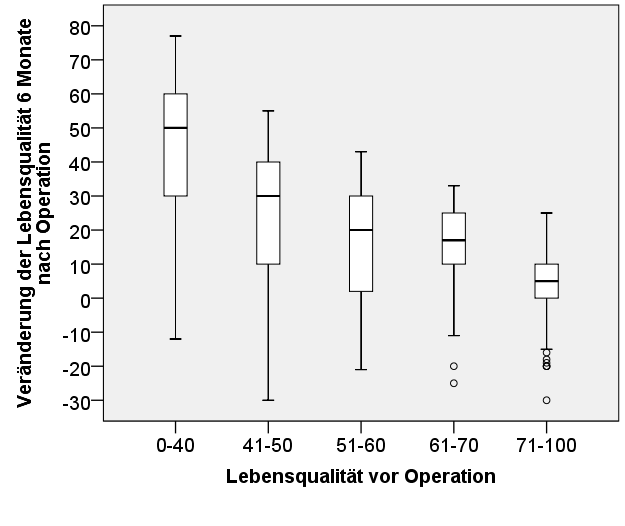
**

**Zufriedenheitsschwelle**

**Zufriedenheitsschwelle**

**Zufriedenheitsschwelle:**

**Zufriedenheit mit dem Ergebnis der Operation 6 Monate nach OP wenn auf einer Skala von 0 - 10 entweder 9 oder 10 angekreuzt wird.**
